# Supplementary material for: Impact of tiny targets on Glossina fuscipes quanzensis, the primary vector of human African trypanosomiasis in the Democratic Republic of the Congo
Source: PLoS Negl Trop Dis. 2020 Oct 16;14(10):e0008270. doi: 10.1371/journal.pntd.0008270 (PMC7608941; doi:10.1371/journal.pntd.0008270)
Supplement: S2 Table — The top five variables with regards to relative importance are shaded in green. Note that (^1) and (^2) in the LASSO column indicate whether the variable was considered as a linear or quadratic term in the model respectively. (DOCX) [file pntd.0008270.s002.docx]

**Table S2:** Environmental variables considered in the boosted regression tree and regularised regression models, plus their relative importance in final models (scaled out of 100). The top five variables with regards to relative importance are shaded in green. Note that (^1) and (^2) in the LASSO column indicate whether the variable was considered as a linear or quadratic term in the model respectively.

| Variable | Variant | Variable importance  (scaled out of 100) | |
| --- | --- | --- | --- |
|  |  | BRT | LASSO |
| Elevation | Elevation | 11 | 19 (^1) |
|  | Ruggedness index | 13 | 43 (^1)  0 (^2) |
| Slope | Mean topographical wetness index (TWI) | 46 | 0 (^1)  100 (^2) |
|  | Minimum TWI | 0 | 18 (^1)  16 (^2) |
|  | Maximum TWI | 100 | 0 (^1)  0 (^2) |
|  | Standard deviation within 350m radius | 12 | 0 (^1)  0 (^2) |
| River network | Vertical distance to nearest point on the river network | 32 | 0 (^1)  0 (^2) |
|  | Euclidean distance to nearest point on the river network | 0 | 0 (^1)  0 (^2) |
|  | Length of river within 350m radius | 25 | 0 (^1)  0 (^2) |
| Land cover | Percentage of forest cells within 350m | 83 | 28 (^1)  65 (^2) |
|  | Percentage of shrub cells within 350m | 3 | 0 (^1)  29 (^2) |
|  | Percentage of water cells within 350m | 7 | 2 (^1)  0 (^2) |
|  | Euclidean distance to nearest water cell | 0 | 0 (^1)  0 (^2) |
| Enhanced Vegetation Index | Mean | 5 | 0 (^1)  0 (^2) |
|  | Maximum | 12 | 0 (^1)  0 (^2) |
|  | Range | 34 | 72 (^1)  0 (^2) |
| Land surface temperature | Land surface temperature | 95 | 0 (^2)  62 (^2) |
