# Supplementary material for: Impact of tiny targets on Glossina fuscipes quanzensis, the primary vector of human African trypanosomiasis in the Democratic Republic of the Congo
Source: PLoS Negl Trop Dis. 2020 Oct 16;14(10):e0008270. doi: 10.1371/journal.pntd.0008270 (PMC7608941; doi:10.1371/journal.pntd.0008270)
Supplement: S3 Table — (DOCX) [file pntd.0008270.s003.docx]

**Table S3:** GLMs and GLMMs fitted to the sentinel site data and their corresponding likelihood and AIC values

| Model ID | Fixed Effects | | Random Effects | df | Log-likelihood | AIC |
| --- | --- | --- | --- | --- | --- | --- |
|  | Additive | Interactions |  |  |  |  |
| A1 | Habitat suitability |  |  | 3 | -588.5 | 1183 |
| R1.1 | Habitat suitability |  | Cluster | 4 | -568.7 | 1145 |
| R1.2 | Habitat suitability |  | Cluster, Site | 5 | -567.3 | 1145 |
|  |  |  |  |  |  |  |
| A2 | In/Out/Edge, Pre/Post | In/Out/Edge* Pre/Post |  | 7 | -509.6 | 1033 |
| A3 | In/Out, Pre/Post | In/Out* Pre/Post |  | 5 | -511.9 | 1033 |
|  |  |  |  |  |  |  |
| R2.1 | In/Out/Edge, Pre/Post | In/Out/Edge* Pre/Post | Cluster |  |  |  |
| R2.2 | In/Out/Edge, Pre/Post | In/Out/Edge* Pre/Post | Cluster, Site |  |  |  |
|  |  |  |  |  |  |  |
| R3.1 | In/Out, Pre/Post | In/Out* Pre/Post | Cluster | 6 | -501.3 | 1014 |
| R3.2 | In/Out, Pre/Post | In/Out* Pre/Post | Cluster, Site | 7 | -498.5 | 1012 |
|  |  |  |  |  |  |  |
| A4 | In/Out/Edge, Intervention length | In/Out/Edge*Intervention length |  | 10 | -502.9 | 1026 |
| A5 | In/Out, Intervention length | In/Out*Intervention length |  | 7 | -508.6 | 1031 |
|  |  |  |  |  |  |  |
| R4.1 | In/Out/Edge, Intervention length | In/Out/Edge*Intervention length | Cluster | 11 | -492.2 | 1006 |
| R4.2 | In/Out/Edge, Intervention length | In/Out/Edge*Intervention length | Cluster, Site | 12 | -489.5 | 1003 |
|  |  |  |  |  |  |  |
| R5.1 | In/Out, Intervention length | In/Out *Intervention length | Cluster | 8 | -496.5 | 1009 |
| R5.2 | In/Out, Intervention length | In/Out *Intervention length | Cluster, Site | 9 | -494.0 | 1006 |
|  |  |  |  |  |  |  |
| A6 | In/Out/Edge, Intervention length, season | In/Out/Edge*Intervention length |  | 11 | -501.5 | 1025 |
|  |  |  |  |  |  |  |
| R6.1 | In/Out/Edge, Intervention length, season | In/Out/Edge*Intervention length | Cluster | 12 | -489.4 | 1003 |
| R6.2 | In/Out/Edge, Intervention length, season | In/Out/Edge*Intervention length | Cluster, Site | 13 | -486.8 | 1000 |
|  |  |  |  |  |  |  |
| A7 | In/Out/Edge, Intervention length, season | In/Out/Edge*Intervention length, habitat*Pre/post intervention |  | 13 | -491.8 | 1010 |
|  |  |  |  |  |  |  |
| R7.1 | In/Out/Edge, Intervention length, season | In/Out/Edge*Intervention length, habitat*Pre/post intervention | Cluster | 14 | -474.4 | 977 |
| R7.2 | In/Out/Edge, Intervention length, season | In/Out/Edge*Intervention length, habitat*Pre/post intervention | Cluster, Site | 15 | -467.1 | 964 |
