# Supplementary material for: Impact of tiny targets on Glossina fuscipes quanzensis, the primary vector of human African trypanosomiasis in the Democratic Republic of the Congo
Source: PLoS Negl Trop Dis. 2020 Oct 16;14(10):e0008270. doi: 10.1371/journal.pntd.0008270 (PMC7608941; doi:10.1371/journal.pntd.0008270)
Supplement: S1 Fig — (DOCX) [file pntd.0008270.s004.docx]

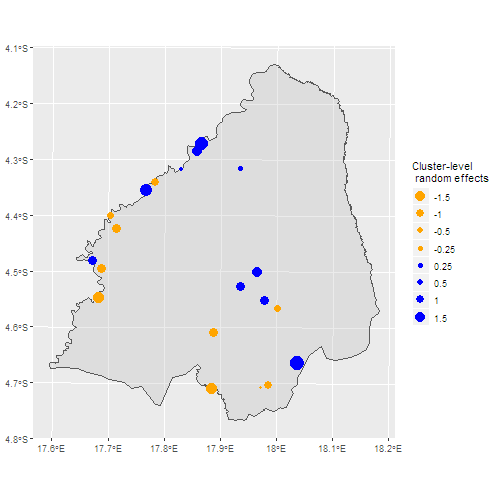


**Figure S1**: Conditional modes of the cluster-level random effects obtained from the final fitted model
