# Supplementary material for: Impact of tiny targets on Glossina fuscipes quanzensis, the primary vector of human African trypanosomiasis in the Democratic Republic of the Congo
Source: PLoS Negl Trop Dis. 2020 Oct 16;14(10):e0008270. doi: 10.1371/journal.pntd.0008270 (PMC7608941; doi:10.1371/journal.pntd.0008270)
Supplement: Attachment — Submitted filename: PNTD-D-20-00527_reviewer_Edits.pdf [file pntd.0008270.s005.pdf]

Key words: habitat suitability, tsetse, vector control, boosted regression trees, remote sensing, human African trypanosomiasis

Introduction

Human African trypanosomiasis (HAT), commonly called sleeping sickness, is a neglected tropical disease caused by sub-species of *Trypanosoma brucei* transmitted by tsetse flies (*Glossina*). In Central and West Africa, HAT is caused by *T. b. gambiense* (Gambian HAT, g-HAT) transmitted by riverine species of tsetse, particularly subspecies *G. palpalis* and *G. fuscipes*. In East and Southern Africa, the disease is caused by *T. b. rhodesiense* (Rhodesian HAT, r-HAT) transmitted by savanna tsetse such as *G. morsitans* and *G. pallidipes*. Both forms of the disease are generally fatal if untreated.

WHO aims to eliminate HAT as a public health problem by 2020 and interrupt all transmission by 2030. The 2020 goal includes the aims of reducing global incidence of HAT to 'fewer than 2000 cases reported globally' and 'gambiense HAT incidence to less than 1 new case per 10,000 population at risk, in at least 90% of foci' [1]. In the period 1990 – 2018, there were 455,086 reported cases of HAT; most (440,055/455,086=96.7%) were cases of Gambian HAT and of these, two-thirds (301,819/440,055=68.6%) were in the Democratic Republic of the Congo (DRC) [2]. During this period, global incidence of g-HAT has declined from a peak of 37,385 cases/year in 1998 to 953 in 2018, the lowest number of HAT cases reported in the history of g-HAT surveillance. The annual incidence in DRC shows a similar trend with cases declining from 26,318 to 660 in the period 1998-2018. The decline globally and in DRC is principally due to large-scale active screening and treatment of the human population [3].

The rapid and sustained decline in the number of cases reported globally means that the 2020 goal of <2,000 cases being reported annually was achieved in 2016 (1,768 cases reported) and has continued to decline year on year. However, there will still be some important foci where HAT is predicted to persist for decades. Analyses of interventions conducted in parts of the former Provinces of Bandundu and Equateur in DRC [4,5] suggest that active screening and treatment will not reduce incidence to <1 case/10,000 people in some Health Zones before 2020. Their analyses suggested however that the elimination goals could be achieved in these Health Zones by adding vector control to the current practice of actively screening the human population [6].

Efforts to control populations of riverine tsetse which transmit *T. b. gambiense* has not formed an important part of efforts against g-HAT. Vector control was not widely used in DRC because the methods available were prohibitively expensive, difficult to apply and/or ineffective. In the past decade however, two simple and cost-effective methods effective against riverine vectors of g-HAT have been developed. First, in areas where cattle are present in sufficient densities (>10 animals/km^2), tsetse can be controlled by treating cattle with pyrethroids [7][8]. Second, where cattle are absent or sparse (<10 animals/km^2), pyrethroid-treated targets, which simulate mammal hosts, can be deployed along the banks of rivers and other water bodies where tsetse concentrate [9–11]. Tsetse are attracted to them and then contact the cattle or targets and in so doing pick up a lethal dose of insecticide. Imposing a modest daily mortality of ≥4% on the female population can eliminate isolated populations of tsetse [12], and reduce densities of non-isolated populations by ~90% [9].

In the past decade, 'Tiny Targets', have been successfully used to control tsetse in g-HAT foci in Uganda [9], Guinea [10] and Chad [11]. Tiny Targets comprise small (25 cm high x 50 cm wide) panels of blue polyester with a flanking panel of black polyethylene netting, both impregnated with deltamethrin insecticide. Tsetse are attracted to the blue panel and as they approach the target they

single time point adequately represented the dry season across multiple years. It was not possible to obtain sufficiently cloud-free imagery for the wet season from the Sentinel-2 satellite and as such no wet season habitat suitability models were derived. It was noted that fly abundance was generally lower in the wet season compared to the dry season for reasons not well understood. A number of potential factors may contribute to the reduction. First, the area of suitable habitat may expand during the wet season leading to an overall reduction in density as flies disperse. Second, the saturated soil may be less suitable for puparia. Third, localised and transient reductions in the temperature associated with rainfall may reduce tsetse activity and hence the numbers attracted to traps. Further investigations are required to test these hypotheses and generate new ones.

Evaluation of the impact of Tiny Targets on tsetse distribution

The catches of tsetse prior to the deployment of Tiny Targets were generally low relative to other areas where sleeping sickness occurs and *G. fuscipes* is the main vector. For instance, in the West Nile focus of Uganda, daily catches of *G. f. fuscipes* were ~2.7 flies/trap (ranging from 0 – 144) [9] compared to 0.7 tsetse/trap (ranging from 0 – 19.5) in the present study. This low apparent density is unexpected given that the high incidence of g-HAT in Yasa Bonga [4]. The low catches of *G. f. quanzensis* in the current study may reflect differences in the sampling efficiency of pyramidal traps for *G. f. quanzensis* and *G. f. fuscipes*. There are no data to allow us to estimate any such difference but studies of other species of tsetse show that there can be large inter-specific differences in sampling efficiency [30]. Alternatively, *G. f. quanzensis* may be a more effective vector of *T. b. gambiensis* by, for example, taking a higher proportion of bloodmeals from humans.

Nonetheless, we were still able to observe that Tiny Targets have a large effect on the fly population in areas close to the intervention (<500m) such that within three months of the targets being deployed catches decreased by >85% compared to their pre-intervention level and remained so throughout the monitoring period. This compares to similar declines for *G. f. fuscipes* in Uganda but is not as marked as that for *G. f. fuscipes* in Chad [11] . The effect diminishes with distance although some benefits are seen in traps situated 500m-5km from targets. This distance effect could not however be fully investigated due to the distribution of traps with respect to distance from targets as few traps were placed far from targets, hence further investigation is needed.

In Yasa Bonga, large numbers (>9,000/deployment) of Tiny Targets were deployed rapidly (~10 days/deployment) on a biannual basis by locally-recruited people operating from canoes. The personnel were not 'professional' staff from a vector control department as at the time of the intervention the vector control staff of the PNLTHA was comprised of only two entomologists, both of whom were based in Kinshasa. As a consequence of this capacity gap, vector control teams were entirely made up of people recruited from communities in the intervention area and using canoes hired from local boat owners. Our work in DRC shows that Tiny Targets provide a method of tsetse control that can be implemented by local people with a small (~1 day) amount of training and ongoing supervision. Moreover, Tiny Targets exerted their greatest impact in areas close (<500m) to where they were deployed. This local effect is likely to strengthen the public perception that Tiny Targets have an impact. Their simplicity and obvious impact suggest that they might therefore be used in some form of community-based approach. However, rapid and regular deployment over large areas, including sections of sparsely inhabited rivers where tsetse are present, means that we caution strongly against the simple assumption that providing local communities with Tiny Targets and a minimum of training will lead to effective control of tsetse. In the present study, the project recruited and paid people from the villages, hired pirogues, provided instruction and supervision of target deployment, and monitored where targets were deployed and their impact on tsetse. These elements were essential to the success of the intervention. Further research is required to assess how local
