# Supplementary material for: Impact of tiny targets on Glossina fuscipes quanzensis, the primary vector of human African trypanosomiasis in the Democratic Republic of the Congo
Source: PLoS Negl Trop Dis. 2020 Oct 16;14(10):e0008270. doi: 10.1371/journal.pntd.0008270 (PMC7608941; doi:10.1371/journal.pntd.0008270)
Supplement: Attachment — Submitted filename: ResponseReviewersLetter.docx [file pntd.0008270.s006.docx]

6th August 2020

Dear PLoSNTDs,

**Re:** Impact of Tiny Targets on *Glossina fuscipes quanzensis*, the primary vector of Human African Trypanosomiasis in the Democratic Republic of Congo

I am pleased to resubmit the revised version of the above paper. Thank you to the reviewers for their comments. I have included our response to each of the points raised below.

**Editorial and Data Presentation Modifications?**

Reviewer #1:

1. Reference is made to 1,234 unique locations in this manuscript as well as s1 T=[table 1]. It would be good for the authors to explain what was unique about these locations and how they were selected for inclusion in this study
2. It would very much help the readers if the authors produced raster [5 km resolution] maps using average FTD data for each trap for the periods; before the intervention; 0-2 months, 3-5 months and >5 months. These visual rendering would help the readers be able to palpate the effect of tinny targets by assessing if; the targets suppress tsetse populations across the entire terrain of the intervention area of if they suppress tsetse population and they shrink into small hot-spot tsetse subpopulations.
3. The authors should consider the possibility of including a discussion point and or a simulation of how many intervention months are required for tiny targets to eliminate tsetse [at least for starters they can simulate isolated tsetse populations] in such a setting and if it is possible they could simulate non- isolated tsetse population with re-infestation prevented by barriers. This suggestion is discretionary!

**Response**

1. ‘Unique’ in this sense does not mean there is anything special about these locations, but rather the term is used to make the point that samples were taken at 1,234 individual locations. We have changed the text to either stating ‘1,234 locations’ or ‘1,234 individual locations’ to remove any ambiguity. We have also clarified how sampling sites were selected in the methods section i.e.

“*Data were collected over multiple field surveys, with the locations of these traps being driven by factors specific to each field visit i.e. exploratory surveys to determine whether flies could be found in specific environments (along stretches of river, around fishponds, in villages). Environmental features of interest were identified using remotely sensed data, whereas specific sampling locations within these environments were selected according to their accessibility, determined using local knowledge and maps of the area.”*

2. We appreciate the suggestion of using raster maps to look at FTD for each period. Unfortunately, neither the temporal inconsistency of the pre-intervention data (data collected over multiple field visits over the three year period) nor the geographical sparsity of the monitoring trap data (yellow triangles in Fig. 1) supports this kind of output. Additional data would be required to demonstrate the effect the reviewer has described.
3. We agree that it would be useful to model the impact of Tiny Targets on populations of tsetse and we have done some preliminary work towards this (Tirados et al., 2015; Vale et al 2015) We are currently developing mathematical and simulation models of the impact of insecticide-treated targets and cattle on tsetse populations but this work is outside the scope of this study. Epidemiological models of g-HAT in Yasa Bonga (Rock et al, 2017) suggest that interruption of transmission can be achieved without eliminating tsetse; reducing the abundance of tsetse by 90% is predicted to be sufficient. We have therefore inserted the following sentence into the Discussion:

“*Epidemiological models of g-HAT in Yasa Bonga (Rock et al, 2017) suggest that interruption of transmission can be achieved by reducing the abundance of tsetse by >60%.”*

**Summary and General Comments**

*Reviewer #2*

1. Although the hypothesis is clearly stated, the objectives are not clearly defined.

**Response**: We have amended the last few sentences of the Introduction to clarify the objectives of the study:

“*A series of large-scale entomological surveys of G. f. quanzensis in Yasa Bonga and its neighbouring health zones of Masi Manimba and Mosango (Figure 1) were conducted to capture information on the distribution of tsetse throughout the area both prior to and following the deployment of targets. and used remotely-sensed data to produce a map of potential tsetse habitat for this important vector, which guided the deployment of targets and allowed us to assess their impact. The objectives of this study were to (a) develop tsetse habitat suitability maps of the area using the pre-intervention (exploratory) entomological surveys combined with remotely sensed data, and (b) combined these maps with data from the subsequent post-intervention (monitoring) surveys to measure the impact of the intervention*.”

2. The study sites are not well described. As a vector-borne diseases and targeting the vectors, it will be important to provide some biotic and abiotic factors characterizing the biotopes where the traps were put during pre-intervention and the monitoring.

**Response**: We have updated the description of the study area in the Methods section to include information on the environment where traps were set.

“*The Yasa Bonga Health Zone is a largely rural area, the population is engaged in cultivation, particularly cassava, and livestock are only present in low numbers. The Health Zone includes three large rivers (Lukula, Kafi, Inzia) and many of the tributaries of these rivers have been altered to form fish ponds. Along the banks of the water courses are bands of dense riverine vegetation including Dalbergia glandulosa, Julbernardia spp, Garcinia kola, Stereospermum kunthianthum, which form the natural habitat for tsetse. Humans and reptiles are likely the main hosts across the Health Zone,* *with domestic pigs in some villages also being of importance*.”

3. No real entomological data is presented in this manuscript.

**Response:** It is unclear to the authors what is meant by this statement. The entomological data included in this paper consists of the number and locations of tsetse captured using pyramidal traps throughout an area of sleeping sickness transmission. We focus on the geographical distribution of flies within this area prior to and following the introduction of an intervention, developing models based on the likelihood that flies are present in an area, and models of fly abundance. The entomological data used in this study has been provided with the submission (Table S1), and plotted in Figures 2-4. If this is not sufficient, could the reviewer please clarify what they mean by ‘entomological data’ in this context?

4. It is mentioned that “The sex and species of collected tsetse were identified morphologically and then stored in 100% ethanol. Subsamples were subsequently analysed at the Liverpool School of Tropical Medicine (LSTM) to confirm species.” There is no data on sex and tsetse species. Where are results of analyses performed at LSTM? How many flies were analyzed at LSTM?

**Response**: Given that it was not considered necessary to use this information to address our objectives, we have removed this statement as it is not relevant.

5. How many flies were collected during the different phases? The authors mentioned that vector control was performed the first year of the study, and the operations were expanded subsequent years. The results section do not provide data related to these control operation: number of flies captured et each time, and the reduction rates

**Response:** Tsetse data were not all collected simultaneously, but rather it was a staggered process. Figure 1 illustrates the progression of the intervention, with pre-intervention studies being colour coded by year. We have amended the text in the Methods section to clarify that these surveys were collected over multiple field visits. Figure 1 also demonstrates the year in which the intervention was introduced. We have updated the caption of this figure to clarify this.

*“**Figure 1: Locations of the 1,234 tsetse monitoring sites at which tsetse flies were collected between January 2015 – December 2017. Pre-intervention sites i.e. sites where data were collected prior to or unaffected by the intervention are triangles colour-coded by year of collection. Monitoring sites i.e. sites sampled immediately prior to the intervention, then repeatedly at regular intervals following the intervention are prepresented by circles. The river networks across which Tiny Targets have been deployed is also presented, colour-coded colour-coded according to the year in which the intervention was first introduced.”*

Table 2 presents a summary of the data that have been collected. This table shows the number of traps deployed, trapping days, flies caught and flies/trap/day by season, regardless of year of collection. This demonstrates the difference in rates between pre-intervention traps (labelled ‘unaffected by the intervention’) and post-intervention traps, split into two groups according to their distance from the targets. We have amended the table caption to clarify this:

*“Table 2: Summary of recorded tsetse data by intervention status and season. Intervention status consists of three categories: (1) traps are considered unaffected by the intervention if they were deployed prior to the intervention or were placed more than 5km away from recently deployed targets, (2) traps were placed 500m-5km from a recently deployed target, (3) traps were placed <500m from a recently deployed target. Data from 2015-2017 are pooled by wet (September – May) and dry (June-August) season.”*

Due to the design of the study we do not believe there is much value in breaking down these summaries down further. Data for all 2474 trapping records are available in the SI, so should this be of interest to the reader then this information is available to them.

6. On which criteria the 1,234 unique locations were selected for pre-intervention surveys of tsetse flies? How many flies were collected? These unique locations are not appearing on Figure 1. What did the authors refer to exploratory in the legend of figure 1? What do they mean by started (legend of figure 1)? Do they refer to areas where the tiny targets were deployed?

**Response**: We have now provided more detail on the selection of the 1,234 individual sampling sites in the Methods section i.e.

“Data were collected over multiple field surveys conducted in 2015-2017, with the locations of these traps being driven by factors specific to each field visit i.e. exploratory surveys to determine whether flies could be found in specific environments (along stretches of river, around fishponds, in villages). Environmental features of interest were identified using remotely sensed data, whereas specific sampling locations within these environments were selected according to their accessibility, determined using local knowledge and maps of the area. ”

The total number of flies collected across these sites (2,474 records) was 837. This number is contained in Table 2, and it has now also been added to the Results section:

“*As of 13^th^ September 2017 there were 2,474 trap monitoring data records at 1,234 locations (1,055 in Yasa Bonga, 110 in Masi Manimba and 69 in Mosango), resulting in 837 flies being captured over 4,952 trapping days.”*

All of the 1,234 locations appear in Figure 1. As the trapping sites are often grouped together, many of the points overlap so it is not possible to see all of the individual symbols representing each site. The caption for Figure 1 has been updated to explain the meaning of ‘exploratory’ and ‘started’.

7. Why in some parts of figure 1, the exploratory of different years occurred in the same place?

**Response**: Figure 1 has been modified to display “pre-intervention” rather than ‘exploratory’ sites and the caption has been updated accordingly.

8. What are the links between pre-intervention and the deployment of Tiny targets? Do results of pre-intervention operations guided the deployment of Tiny targets?

**Response:** A more detailed description of pre-intervention sites is included in the methods. Further, it is stated in the Methods that “*Based on results from the pre-intervention surveys of tsetse, we selected 23 locations which produced the largest tsetse catches to monitor the impact of targets.”*

9. Some legends of figure 2 are not appearing in this figure.

**Response**: We are unclear what components do not appear in the figure and have therefore been unable to address this comment.

10. The legend related to tsetse count of figure 2 is not relevant. It can be seen as presence or absence of tsetse. It will be more convenient to present the relative density of tsetse flies

**Response**: The habitat suitability map is derived from presence/absence, however we have updated Figure 2 so that fly density rather than presence/absence is displayed. The caption for Figure 2 has also been updated.

“*A map of habitat suitability obtained using a boosted regression tree approach. The fly counts (flies/trap/day) of each of the 332 trap sites used to fit the model is also displayed.”*

11. From legends of figure 2, different estimated habitat susceptibility have been identified. What are the main characteristics of each habitat?

**Response**: The habitat suitability score is a proportion that has been derived by fitting a model to the presence/absence data. Suitability in this instance was largely driven by remotely sensed information obtained on forest cover, enhanced vegetation index, topographical water index and land surface temperature. This information is presented in the results section, with supporting data being provided in Table S2:

*“The relative importance of each of the environmental variables with respect to their contribution to the final BRT and LASSO models is presented in Table S2 of the SI. In both models the percentage of forest cover within a 350m radius, the range of EVI within a 350m radius, the mean topographical water index (TWI) within a 350m radius and land surface temperature are important contributors to the model. Maximum TWI is also identified as important to the fit in the BRT model, whereas ruggedness is selected for the LASSO model.”*

12. The authors mentioned in the discussion that “This low apparent density is unexpected given that the high incidence of g-HAT in Yasa Bonga [4]”. They tried to link tsetse density with the disease prevalence in humans. However, they do not remember that the transmission of trypanosomes does not depend entirely of tsetse density, but on the contact frequency between tsetse and vertebrate hosts. The tsetse density may also depend on the availability of vertebrate hosts. These two parameters have not been mentioned in this study.

**Response:** We have included the following sentences into our Discussion:

“*While the abundance of tsetse is an important factor in disease risk, transmission depends on, amongst other things, abundance of hosts at points where humans and/or animal hosts and tsetse contact each other. Our study did not include fine scale analyses of the abundance and movement of humans and animals and such work might explain why incidence of g-HAT is high in places where catches of tsetse are low*.”

13. It is mentioned in the results section that “At baseline, there was no significant difference in the number of flies caught in traps set in areas with differing intervention status i.e. areas within the intervention area (‘in’), well outside of the intervention area (‘out’) and intermediate areas (‘edge’).” Where are these data?

**Response**: This results was presented in Table 7, and the text has now been supplemented to present this result:

*“At baseline, there was no significant difference in the number of flies caught in traps set in areas with differing intervention status i.e. areas within the intervention area (‘in’, adjusted log-RR = 0.65, 95% CI = [-1.3275, 3.7609]), well outside of the intervention area (‘out’, reference level in the model) and intermediate areas (‘edge’, adjusted log-RR = 2.05, 95% CI = [-0.9337, 4.4011]).”*

14. It is also mentioned that “Habitat suitability is significantly and positively associated with fly catches prior to the introduction of the intervention”. What type of habitat are the authors referring to?

**Response**: This refers to the habitat suitability value (a proportion between 0 and 1) that has been derived from fitting the boosted regression tree model to the presence/absence data. Additional text has been included in the results to clarify this:

*“Covariates considered in the GLMM were therefore the proximity of the intervention (two-level or three-level factor), time since the intervention was initiated (two-level or three-level factor), habitat suitability derived from the BRT model and season (wet/dry).”*

15. Data of all tables are difficult to understand. The authors must give exactly what the data of each column are referring to. They must also include the footnotes for each table

**Response:** The caption for Table 2 has been amended as previously described. Table 3 has been amended as follows:

*“Table 3: Summary of the number of pre-intervention trap deployments by year and season (wet/dry). Date from these sites were used to develop the habitat suitability maps. Unique locations refers to the number of locations where at least one trap was deployed over the three year period. Positive sites are those where at least one fly was captured during any deployment.”*

The layout of Table 4 has been amended to make it simpler to understand:

*Table 4: ROC-AUC and Brier score of the final fitted BRT and LASSO models plus the median andrange of the ROC-AUC and Brier score for the 100 cross-validated predictions.*

|  |  |  | ***Brier Score*** | ***ROC-AUC*** |
| --- | --- | --- | --- | --- |
| ***Final model*** | ***BRT*** |  | *0.0975* | *0.9402* |
|  | ***LASSO*** |  | *0.1276* | *0.8089* |
|  |  |  |  |  |
|  |  |  |  |  |
| ***Cross-validation*** | ***BRT*** | *Median* | *0.1410* | *0.7583* |
|  |  | *Range* | *0.1336 – 0.1491* | *0.7207 – 0.7843* |
|  | ***LASSO*** | *Median* | *0.1393* | *0.7491* |
|  |  | *Range* | *0.1356 – 0.1575* | *0.7071 – 0.7727* |

The caption for Table 5 has been amended as follows:

*“Table 5: Summaries of trap data (number of traps deployed and flies/trap/day) plus estimated dry season habitat suitability (median and interquartile range extracted from the BRT model) by distance to nearest Tiny Target”*

The caption for Table 6 has been amended as follows:

*“Table 6:* *Summary of fly data (number of traps deployed, number and percentage of traps where tsetse were present and flies/trap/day) by the number of months since the nearest target was deployed (0-2, 3-5, 6+ or pre-intervention/never) and season (wet/dry).”*

The caption for Table 7 has been amended as follows:

*“Table 7: Model output (adjusted log-relative risk and corresponding 95% confidence intervals) for each component of the final selected GLMM fitted to the sentinel site data”*

Reviewer #3:

The manuscript has good research and has been well articulated. I have just a few recommendations to the authors:

1. It should be indicated that much as riverine tsetse is in west and central Africa, it is also present in some parts of East Africa, particularly the Lake Victoria and River Nile Basin.

**Response**: We have changed the first paragraph so that it now reads:

“*particularly subspecies of G. palpalis in west Africa and G. fuscipes in Central Africa and the Lake Victoria basin and upper reaches of the river Nile in East Africa.* “

2. The findings about seasonal tsetse distribution and abundance need to be discussed in relation to previous studies.

**Response**: We have amended the Discussion section as follows:

“*In this study we found that there were modest seasonal variations in catches of tsetse with a mean of 0.04 tsetse/trap/day in the wet season (Sep-May) compared to 0.1 tsetse/trap/day in the dry season (June-August). De Deken et al. (2005) also reported that catches of G. f. quanzensis at sites in the Kisenso District of DRC were generally low with numbers being greatest in December-January (~0.7 tsetse/trap/day) during the wet season (defined as October-April in their study) and lowest (~0.5 tsetse/trap/day) at the start of the dry season (May to July). Tirados et al. (2015) also found that seasonal fluctuations in the catches of G. f. fuscipes in Uganda were relatively small (0.7-3.9 tsetse/trap/day) and studies of the population genetics of G. f. fuscipes in Uganda also suggest that populations are temporally stable (Echodu et al., 2011, Hyseni et al., 2012, Opiro et al., 2016). This temporal stability in populations of G. f. fuscipes contrasts with the strong seasonal fluctuations displayed by savanna species such as G. morsitans morsitans and G. pallidipes where there are >10-fold differences in catches between seasons (Hargrove & Vale, 1980). The stability of G. fuscipes populations is probably related to the more equable seasons found in central Africa where hot dry seasons are shorter and less extreme.*”

Sincerely,

Dr Michelle Stanton, PhD

Lecturer
